# Preliminary evidence that fatigue contributes to anhedonia in stable individuals diagnosed with schizophrenia

**DOI:** 10.3389/fpsyt.2023.1098932

**Published:** 2023-01-27

**Authors:** Yasmine Laraki, Sophie Bayard, Amandine Decombe, Delphine Capdevielle, Stéphane Raffard

**Affiliations:** ^1^University Department of Adult Psychiatry, CHU Montpellier, University of Montpellier, Hôpital la Colombière, Montpellier, France; ^2^Université Paul Valéry Montpellier 3, EPSYLON EA, Bouisson Bertrand, Montpellier, France; ^3^IGF, University of Montpellier, CNRS, INSERM, Montpellier, France

**Keywords:** fatigue impact, anhedonia, depression, schizophrenia, primary and secondary negative symptoms

## Abstract

**Objectives:**

Anhedonia and fatigue are trans-diagnostic symptoms commonly observed in schizophrenia. Anhedonia is a core negative symptom with a strong relationship with depression and is associated with diminished global functioning. Similarly, fatigue is also associated to depression and research across psychiatric illnesses indicate that fatigue may persist even when primary symptoms are treated. Although fatigue is common in people diagnosed with schizophrenia, it is under studied within this population. The objective of this exploratory study was to investigate the association of fatigue and anhedonia by controlling for depression in a sample of individuals diagnosed with schizophrenia.

**Method:**

Fifty-one stable individuals diagnosed with schizophrenia from the University Department of Adult Psychiatry in Montpellier took part in this study. Participants completed questionnaires on fatigue impact and depression, and were assessed for symptom severity. Following data collection, statistical analyses were conducted in order to explore associations between clinical variables and fatigue impact. Based on the results obtained, a hierarchical linear regression was conducted in order to investigate whether fatigue impact contributed to the variance of negative symptoms.

**Results:**

The hierarchical linear regression indicated that when controlling for depression, fatigue impact contributes to ~20% of the variance of anhedonia. Together the social impact of fatigue and depression contribute to 24% of the variation of anhedonia.

**Conclusion:**

To the best of our knowledge, this exploratory study is the first to investigate and show that fatigue impact may contribute to anhedonia. We recommend further research to investigate fatigue, its impact on symptomatology, and better categorization of negative symptoms in hopes of developing targeted fatigue treatment interventions.

## Introduction

Negative symptoms (NS) of schizophrenia are defined as a reduction or absence of normal behaviors and functions like interest, motivation, expressed emotions or speech (1). Recent evidence (2, 3) suggests that five dimensions best reflect the conceptualization of NS: (1) blunted affect, diminished expressions of emotion; (2) alogia, decreased quantity of spoken words; (3) avolition, a reduction in initiating goal-directed behaviors due to diminished motivation; (4) asociality, diminished social interactions due to reduced interest in interpersonal relationships; and (5) anhedonia, diminished experience of pleasure in an activity or anticipated pleasure (1, 4).

The symptom of anhedonia, conceptualized as an inability to experience pleasure and respond to pleasurable stimuli (5) is considered a trans-diagnostic symptom. As such anhedonia is present across different psychiatric disorders including schizophrenia or major depressive disorder (MDD) (4), but also across neurodegenerative conditions such as frontotemporal dementia or Alzheimer’s disease (6). From a neuroanatomical perspective, recent imaging studies have shown that similar brain regions and circuits were implicated with an overlap in neural activations and abnormalities of clinical anhedonia in MDD, schizophrenia and in bipolar disorder (7, 8). Overlapping pathophysiological circuitry abnormalities of clinical anhedonia across different pathologies contribute to the mounting evidence of the trans-diagnostic nature of this symptom (5–7, 9).

Precisely in schizophrenia, anhedonia has been shown to strongly associate with poorer global functioning, and was a strong predictor of reduced quality of life, social and vocational functional outcomes (10). As such anhedonia is considered a debilitating symptom with limited therapeutic effectiveness with the currently available medical treatment options (1). Furthermore, antipsychotic clinical trials have also shown that NS were prominent despite medical treatment, hence showing that medication options show little efficacy on the reduction of NS in general (11–13). Nevertheless, a few studies have shown moderate improvement in NS using pharmaceutical treatments, but these studies involved solely individuals in an acute psychotic illness phase and based on short-term assessments only (14, 15). On the other hand, psychosocial interventions, such as cognitive behavioral therapy (CBT) interventions have shown positive, yet moderate effects on negative symptomatology, and this mostly in terms of reduced apathy and overall motivation (1, 16–18). Although trials are still needed in order to increase evidence for these interventions, the positive effects of CBT remains an area to be further investigated as trials have suggested clinical improvement (1).

Furthermore, one important symptom that has been associated with both anhedonia and depression is the trans-diagnostic symptom of fatigue. Though there is no consensus on the exact definition of fatigue, it is generally defined as a subjective and multidimensional construct of cognitive, physical, and emotional tiredness, or lack of energy (19). This disabling symptom is commonly experienced in general populations, in chronic somatic illnesses as well as in psychiatric illnesses such as in MDD (20), and in schizophrenia (21). Specifically, experiences of chronic fatigue in individuals diagnosed with severe mental illnesses, including schizophrenia, bipolar disorder, and MDD, will have negative repercussions on daily life (22). As such, chronic fatigue in severe mental illnesses acts as an obstacle in initiating or maintaining physical activity (23), and may lead to cognitive difficulties such as lack of concentration, diminished overall functional health (21) and reduced interpersonal relations (24).

In addition, fatigue has also been shown to be closely tied to symptoms of depression (19). For example, Billones et al. (19) exposes a study investigating fatigue in women diagnosed with chronic fatigue syndrome, post cancer, or major depression (25) whereby the authors conclude that fatigue was an indistinguishable symptom from depression. Specifically within schizophrenia, at least one quarter of patients meet the criteria for a clinical depression at some point during the course of illness (26), and these symptoms may be present at different phases including prodromal to psychosis, first-episode psychosis, as well as in chronic patients (27). Though the exact prevalence rate of comorbid depression in schizophrenic populations varies from study to study, the co-existence of depressive symptoms in this population is indisputable. Furthermore, Wigman et al. (28), claim that depression (whether at a symptom or syndrome level) exists within the majority of individuals suffering from schizophrenia. An estimated 50% of schizophrenic patients will have a comorbid episode of depression throughout the course of their illness (27). According to the 2010 Australian national survey, 79.6% of people diagnosed with a psychotic illness suffered from depressive symptoms within their lifetime, and a total of 54.5% within that previous year (29). Moreover, Wigman et al. (28), advise that the high co-presence of psychotic illnesses with mood disorders should be understood as a network of overlapping and reciprocally influencing dimensions. For example, in a systematic review (30), the authors outlined the relationship between negative and depressive symptoms in psychotic patients. Symptoms of anhedonia, avolition and anergia (lacking energy) were common to both negative and depressive symptoms. Though the authors conclude that there is evidence for discrimination between both constructs, there remains a large overlap between these symptom domains (30). Overall, the body of research suggests that depressive symptoms are closely tied with negative symptomatology of schizophrenia (31). Finally, even though anhedonia and depression may show overlap, multiple factor analytic studies have shown that these symptoms form separate constructs (32–34).

To date few studies have investigated fatigue specifically within schizophrenia. Yet, Waters et al. (21) showed that up to 60% of their sample group, composed of 67 participants diagnosed with schizophrenia, reported high levels of fatigue. In another study with 79 participants with a diagnosis of schizophrenia, the authors showed that functional health, defined as an individual’s ability to take part in daily living (both physical and mental capacities), was lowest in individuals experiencing higher levels of fatigue (35). Significant levels of fatigue was also shown to have detrimental effects on daily lives particularly in the domains of mental, social, and general facets of functional health. However, the physical effects of fatigue did not appear to significantly contribute to diminished functional health in this clinical population (21). Overall, in their study, regression analyses demonstrated that gender and fatigue had the largest impact on functional health in their sample (21).

In addition, though research on fatigue in schizophrenia is lacking, few studies have indicated a strong relationship between fatigue and anhedonia in other psychiatric illnesses particularly in MDD (19, 36). Importantly, network analyses in MDD have revealed that depressed mood and fatigue were central and separate symptom clusters, but that both were highly characterized with recurrent connections with anhedonia (36). In a recent review (19), the authors explored the differences between the two constructs. Altogether, 60% of the reviewed articles considered fatigue and anhedonia as distinct constructs and implicating distinct biological underpinnings. Together with depression, these symptoms may overlap through shared mechanisms and behavioral characteristics (19). Overall, the body of literature shows that anhedonia is highly associated with fatigue and depression, and that both fatigue and anhedonia may be an important source of distress and functional limitation.

Though research demonstrates that fatigue is a clinically important symptom, it has yet to be examined within the context of negative symptoms of schizophrenia. Therefore, understanding whether anhedonia is associated to fatigue specifically within the context of schizophrenia remains underexplored. Hence, the objective of this exploratory study was to investigate the independent contribution of fatigue to anhedonia by controlling for symptoms of depression in a sample of clinically stable individuals diagnosed with schizophrenia. Given that fatigue is a subjective and individual experience, we explored the perceived impact of fatigue on functional health. Overall, we were interested in understanding whether the effects of fatigue contributed to the variance of NS, specifically of anhedonia, in a population of stable individuals diagnosed with schizophrenia. Based on the aforementioned evidence, we hypothesized that higher fatigue impact would be positively associated with higher levels of anhedonia, independently of symptoms of depression.

## Materials and methods

### Participants and procedure

A total of 51 outpatients with a diagnosis of schizophrenia were recruited from the University Department of Adult Psychiatry in Montpellier, France between January 2021 and February 2022. All participants had to be aged between 18 and 60 and be fluent in French. Exclusion criteria was a history of a traumatic brain injury, known neurological disorder, and a dependence of a substance (except for cannabis or tobacco). All participants had to have a confirmed diagnosis of a schizophrenia disorder based on the DSM-5 (4) criteria and in a stable phase of illness (no hospitalizations in the past 6 months, and no changes in medication was expected). A diagnosis of schizophrenia was confirmed for each participant based on medical records. After receiving a full explanation of the study, all participants agreeing to take part in the study, gave their informed and written consent. Demographic and treatment dosages (if any) were collected from all participants before administering the measures. Treatment dosages were calculated as chlorpromazine equivalents as well as antipsychotic type (typical, atypical, or a combination).

The hospital’s institutional ethics committee approved this study and was conducted in accordance to the Declaration of Helsinki 1975 standards for human experimentation (Ethical Committee # 202000538).

### Measures

The Fatigue Impact Scale (FIS) (37) is a 40-item self-report questionnaire developed to measure the impact of fatigue on quality of life. Precisely, rather than measuring the level of fatigue *per se*, results on the FIS reflects functional limitation experienced over the past month due to fatigue (38). The French version of the FIS includes the following dimensions: cognitive (10 items), physical (13 items), social (13 items), and psychological (four items) (39). The cognitive dimension reflects the impact of fatigue on concentration memory and organization of thoughts. The physical functioning domain describes the impact of fatigue upon motivation, physical effort and coordination. The social functioning domain reflects the impact of fatigue on social contacts, motivation, and isolation. Finally, the psychological dimension concerns the impact of fatigue on emotions and coping. All items are scored on a 5-point Likert scale, ranging from 0 (it’s always false) to 4 (it’s always true). Total scores range from 0 to 160, with higher scores indicating increased fatigue impact. In this study the FIS had excellent internal consistency (total score, *α* = 0.94).

The Beck’s Depression Inventory II (BDI) (40, 41), a 21-item self-reported instrument, was used to measure presence and level of depressive symptoms. Item response on the BDI range from 0 to 3, with total scores ranging from 0 to 63, whereby higher scores indicate higher severity of depressive symptoms. The French version of the BDI was used in this study and showed high internal consistency coefficient (total score, *α* = 0.87).

The Positive and Negative Symptom Scale (PANSS) (42) is a 30-item observer-rater scale used for measuring symptoms of schizophrenia. This 30-item scale was initially conceptualized based on a three-factor model: positive symptoms (seven items), negative symptoms (seven items), and general psychopathology (16 items). However, recent research suggests that a five-factor model better represents the symptomatology present in this population (43, 44). Such has also been concluded in the French validation of the PANSS demonstrating good psychometric properties using a five-factor model (45, 46). For this study, the five factor model of the PANSS was used (43, 44). Internal consistency for the total score of the PANSS was very good with a Cronbach’s alpha of 0.79.

Negative symptoms was measured using the Scale for the Assessment of Negative Symptoms (SANS) (47). The SANS is a clinician-rated measure composed of 25 items grouped into five dimensions: affective flattening, alogia, avolition-apathy, anhedonia-asociality (and attention). For the purpose of our analyses, NS were analyzed independently as to better capture which symptom dimension most contributed to the impact of fatigue. We have not analyzed the items pertaining to attention, as based on the NIMH research attention is no longer considered one of the five core NS (2). For clarity and transparency, we have named hereafter the subscale of anhedonia/asociality as anhedonia. Indeed the composite score of this subscale is composed of items referring to reductions in interest in both pleasurable activities as well as in social relaitonships (32). Given that a disinterest and reduced experienced pleasure in social relationships is also conceptualized as social anhedonia (48), we used the general term of anhedonia. In this study, the SANS had very strong internal consistency (total score, *α* = 0.90).

### Statistical analysis plan

All data from questionnaires were tested for normality using values for skewness and kurtosis (49). No normal distribution was considered if absolute values were >3 and 10, for skewness and kurtosis, respectively (49). Data normality was confirmed for all variables allowing for parametric tests to be used. We first ran preliminary analyses on clinical variables including means, standard deviations, and variable ranges. Then, associations between variables were explored using two-tailed Pearson’s correlations. In order to avoid Type 1 errors, we conducted Bonferroni corrections by dividing the original *α*-value (set at 0.05) by the number of analyses performed on our variables, resulting in lowering the *p*-value required for significance. In order to investigate associations between continuous and categorical variables, a one-way ANOVA was used with results of the post-hoc comparisons, if a significant *p*-value was obtained. Based on significant correlations, we conducted a hierarchical linear regression to determine whether fatigue impact (and depression) contributed to the variance of NS. Prior to conducting these analyses, we checked for the assumptions of normality, homoscedasticity, linearity, and the absence of multicollinearity for our variables using predicted probability plots, scatterplots, normal distribution of residuals, and the variation inflation factors (VIF), respectively (50). Multicollinearity was tested using the variation inflation factors and tolerance statistics. VIF’s are expected to be below 10 and tolerance statistics above 0.2 (50). When these criteria are satisfied, then we may assume that there are no issues regarding collinearity among our predictors.

All data analyses were performed using IBM Statistical Package for the Social Sciences – version 24.0 with a two-tailed level *α* of 5% (51).

## Results

### Demographic and descriptive statistics of clinical variables

The means, standard deviations, and ranges for demographic and clinical measures are provided in Table 1. There was no significant effect of gender, *t*(49) = − 0.34, *p* = 0.33, as women (*M* = 80.64, *SD* = 22.75) scored similarly to males (*M* = 77.43, *SD* = 28.83) in terms of total fatigue impact (Table 1 near here).

**Table 1:** Demographic and clinical variables for all participants; *n* = 51.

Variable	Mean or %	SD	Range
Demographic variables
Gender (male)	80%	/	/
Age, years	33.14	7.59	22–51
Schooling, years	12.71	2.91	7–23
Clinical variables
Duration of illness, years	10.31	7.27	1–28
FIS total	78.12	27.45	16–144
FIS cognitive	19.29	8.18	3–39
FIS psychological	7.65	4.01	0–15
FIS physical	26.25	9.29	4–42
FIS social role	24.92	10.02	4–48
BDI	13.78	9.59	0–41
PANSS
PANSS positive	14.35	5.83	7–30
PANSS negative	15.55	5.76	6–31
PANSS disorganization	15.12	5.34	9–31
PANSS excitation	11.84	3.16	8–20
PANSS emotional distress	15.84	4.42	8–27
SANS total score	27.69	16.54	2–75
SANS affective flattening	5.90	6.13	0–24
SANS alogia	2.37	3.10	0–11
SANS avolition	5.14	3.11	0–11
SANS anhedonia	6.43	4.33	0–15
Antipsychotic treatment (typical, atypical, combination)	3.9, 54.9, 40.0%	/	/
Antipsychotic dosage (CpzEq)	605.39	590.26	0–2,700

FIS, Fatigue Impact Scale; BDI, Beck’s Depression Inventory; PANSS, Positive and Negative Syndrome Scale; SANS, Scale for the Assessment of Negative Symptoms, note that SANS attention was removed from all analyses; CpzEq, Chlorpromazine equivalent dose.

### Correlation analyses

Relations between fatigue impact and symptomatology were explored using two-tailed Pearson’s correlations (see Table 2). Bonferonni corrections were applied resulting in lowering the *p*-value necessary for significance to 0.0009. Our results indicated that fatigue impact in the social role domain was mostly associated with anhedonia, emotional distress and depression. Physical fatigue impact did not correlate with any of our measured variables. Depression was correlated with cognitive, psychological, and social fatigue impact domains, but not with the physical domain. Depression was most strongly correlated with fatigue impact in the social role domain (*r* = 0.66, *p* < 0.0001). Finally, a one-way ANOVA revealed that there was no statistically significant difference in total fatigue impact scores between at least two of the antipsychotic types (*F*(2,47) = [0.04], *p* = 0.96) categorized as typical (*M* = 77.50, *SD* = 31.82), atypical (*M* = 76.25, *SD* = 29.60), or a combination (*M* = 78.50, *SD* = 26.93). Similarly, no association was found between total fatigue impact and Chlorpromazine equivalent dosages: *r* = −0.05, *p* = 0.18 (Table 2 near here).

**Table 2:** Pearson correlations between variables of interest (using Bonferroni corrections); *n* = 51.

	FIS – cognitive subscale	FIS – psychological subscale	FIS – physical subscale	FIS – social role subscale	FIS – total score
1. BDI	**0.53** *	**0.56** *	0.42	**0.66** *	**0.62** *
2. Positive subscale (PANSS)	−0.03	0.12	0.05	0.13	0.07
3. Negative subscale (PANSS)	0.06	−0.08	−0.00	0.16	0.06
4. Disorganization subscale (PANSS)	0.02	0.04	0.07	0.14	0.09
5. Excitation subscale (PANSS)	−0.16	0.05	0.00	0.12	0.00
6. Emotional distress subscale (PANSS)	0.28	0.40	0.22	**0.50** *	0.40
7. Blunted affect subscale (SANS)	−0.13	−0.15	−0.22	−0.07	−0.17
8. Alogia subscale (SANS)	−0.19	−0.21	−0.25	−0.05	−0.19
9. Anhedonia/asociality subscale (SANS)	0.35	0.13	0.20	**0.49** *	0.37
10. Apathy/avolition subscale (SANS)	0.14	0.16	0.21	0.30	0.24

Significant correlations are set in bold. FIS, Fatigue Impact Scale; BDI, Beck’s Depression Inventory; PANSS, Positive and Negative Symptom Scale using the Van der Gaag model; SANS, Scale for the Assessment of Negative Symptoms.

* The corrected *p*-value for Bonferroni correction is *p*_corr_ < 0.0009.

### Hierarchical regressions

Based on the results obtained on correlational analyses, we conducted a hierarchical regression analysis in order to explore whether the impact of fatigue on the social domain contributed to the variation of anhedonia, independently of depression. According to the aforementioned criteria, there were no issues regarding collinearity. Depression was entered into the first step of the model, and social impact of fatigue was entered in the second step (see Table 3). Depression accounted for 6% of variance in anhedonia [*F*(1,49) = 4.24, *p* = 0.045], and when fatigue impact in the social role domain was entered, both variables explained ~24% of anhedonia variance. Social role fatigue impact explained 20.8% of the variance alone [*F*(2,48) = 7.56, *p* = 0.001] (Table 3 near here).

**Table 3:** Hierarchical regression analyses to examine the influence of depression and social role of fatigue impact on symptoms of Anhedonia.

Step variables	*β*	*t*	*R* ^2^	Δ*R*^2^	*F*	*p*
*Anhedonia*
1. Depression	−0.07	−0.41	0.06	0.08	4.24	0.05*
2. Fatigue impact – Social Role	0.53	3.18	0.21	0.16	10.10	≤0.001*

Anhedonia measured using the composite score of the Anhedonia/Asociality subscale of the Scale for the Assessment of Negative Symptoms; Depression measured using the total score of the Beck’s Depression Inventory, and Fatigue impact measured using the Fatigue Impact Scale – social role subscale

## Discussion

The present exploratory study examined the independent contribution of fatigue impact unto NS, specifically of anhedonia in a population of stable participants diagnosed with schizophrenia. The results obtained in this study are in line with our hypothesis that higher fatigue impact would be positively associated with higher levels of anhedonia and independently of symptoms of depression. After having established associations between fatigue impact and anhedonia, we used hierarchical regression analyses to study the degree to which fatigue impact contributes to the variance of anhedonia. Firstly, results based on the FIS indicated that fatigue impact was highest in the physical and social role domains (and lowest in the psychological domain). Secondly, we demonstrated a clear association between the NS of anhedonia and the social impact of fatigue. Thirdly, and most importantly, based on our hierarchical regression model, our results suggest that the impact of fatigue in the social domain accounts for ~20% of the variance of anhedonia in our sample group.

Similarly to our results, Waters et al. (21) also demonstrated that the effect of fatigue was particularly present in general and social dimensions of functional health, whereas the physical dimensions were less prominent in a sample of individuals diagnosed with schizophrenia. A potential explanation could be that higher levels of anhedonia can disturb an individual’s capacity to predict the pleasure arising from interpersonal relationships (social anhedonia). Due to high levels of fatigue, an individual could further limit interpersonal interactions, and causing to a larger degree fatigue impact in the social domain compared to the other sub-domains researched. Additionally, our results suggest a clear association between depression and fatigue impact. As such, depression correlated with all domains of fatigue impact, though it was most associated with the social role dimension. This indicates that the impact of reduced interpersonal relationships (and interest in social activities) due to fatigue may contribute to a higher degree to the lowered mood in schizophrenia (compared to the other fatigue impact domains).

Most importantly, using hierarchical regressions, our results indicate that 20% of the variance of anhedonia, defined as diminished experiences of pleasure in an activity or anticipated pleasure (1, 4) can be explained by the impact of fatigue in the social domain. In other words, even when controlling for depression, a commonly observed symptom in schizophrenia, our study shows that fatigue plays an important role in the level of anhedonia. To the best of our knowledge, this is the first study to suggest that the impact of fatigue influences the variation of anhedonia in schizophrenia. The current body of literature has previously demonstrated a strong link between depression and anhedonia, but this study provides us with additional information that fatigue impact in the social domain may directly influence the symptom of anhedonia in this clinical population.

Though this study suggests that fatigue impact may influence negative symptomatology present in individuals diagnosed with schizophrenia, whether this link specifically pertains to primary versus secondary negative symptoms remains unexplored. Primary NS refers to symptoms that are intrinsic to the pathology (pertaining to the motivation and expression domains) whereas secondary NS are triggered by various underlying mechanisms including symptoms of depression, side-effects of antipsychotic medication, positive symptoms, anxiety, or other environmental factors (52). For example, an individual experiencing constant threat from paranoid or persecutory ideations, could lead to social withdrawal, exemplifying a secondary effect of positive symptoms. As such, in the case of anhedonia, this symptom may well be of primary or secondary nature. This emotional disturbance is commonly present in early stages of the disease onset (53, 54) indicating that this symptom is not merely present as a consequence of long-term medication usage or depression (32). Undeniably, the ability to distinguish between primary and secondary anhedonia would provide clinicians with valuable clinical insight, and could facilitate the recommendation of appropriate therapeutic treatment options (52, 55). Regrettably, there are currently no validated tools that allow differentiating between primary and secondary anhedonia in schizophrenia.

In addition, studies have shown that despite improvement in primary symptoms of a certain pathology, symptoms of fatigue may persist. For example in a large European epidemiologic study including 1,884 individuals diagnosed with MDD, 73% of the sample reported fatigue as a main symptom (56, 57). In their review, Baldwin et al. (58) demonstrate that fatigue (along with sleepiness) are considered residual symptoms in people diagnosed with MDD who have been treated with antidepressants. In Maurizio et al.’ review (59), similar conclusions were found whereby the presence of residual fatigue in participants diagnosed with MDD was a strong predictor of impaired psychosocial functioning and hindered complete remission. A recent study assessed the level of fatigue in 1063 clinically stable psychiatric participants with either a diagnosis of MDD, bipolar disorder or schizophrenia, during the COVID-19 outbreak (22). The authors of this multi-center cross-sectional study included only stable individuals older than 50 years of age and concluded that prevalence of fatigue was high (47.1% of their sample group) even when primary symptoms of the psychiatric illness was stabilized (22). In addition, though antipsychotic medication has been shown to exacerbate and increase fatigue experienced in this population (24), our results did not show an association between chlorpromazine dosage equivalence (nor anti-psychotic type) and fatigue impact. Overall, these studies along with the results of our study may indicate that symptoms of fatigue in schizophrenia may act as a trigger for secondary NS and even contribute to maintaining other symptoms present in this pathology.

Furthermore, though our results highlight the important role of fatigue, its functional impact in schizophrenia, as well as its close link with anhedonia, the underlying mechanisms of fatigue involved in this pathology is another area that remains underexplored. For example, there is evidence suggesting that symptoms of increased fatigue may be associated with increased metabolic syndrome factors in individuals diagnosed with chronic fatigue syndrome (60). In addition, in a systematic review investigating etiologic mechanisms of fatigue (61), the authors have also suggested a potential influence of dysfunctions in mitochondrial structure in people with chronic fatigue syndrome, multiple sclerosis, HIV and cancer-related fatigue. Despite evidence for biological markers implicated in fatigue in chronic illnesses, these mechanisms have still not been explored specifically within psychotic disorders. Improving our understanding on the origins and potential causes of fatigue in schizophrenia would provide us with valuable information on ameliorating the presence and functional impact of this symptom. Current interventions such as Positive Emotions Program for Schizophrenia (62), has been shown to decrease levels of anhedonia, but to our knowledge no study has tested the efficacy of fatigue interventions in schizophrenia. As such, both CBT interventions targeting better fatigue management (63) and a better understanding of underlying biological markers of fatigue in schizophrenia, may be key in reducing overall level of NS, depression, and burden in this population. Such interventions may well have a large and positive impact on the quality of life and daily living, through diminishing levels of experienced fatigue and better energy management.

## Limits

The first limit to our study is that depression in our sample was measured using Beck’s Depression Inventory (40) rather than using the Calgary Depression Scale for Schizophrenia (CDSS) (64). Similarly, instead of using the PANSS (42) or the SANS (47), the use of the Clinical Assessment Interview for Negative Symptoms (65–67) or Brief Negative Symptom Scale (68) as main measures of NS, could have rendered higher accuracy in our results. Nevertheless, these tools had yet to be validated in French when constructing this study. We are aware, that using tools specifically validated in our clinical population, would have been a better representation of symptomatology (69). Based on BDI standards, another limitation to our study is that our clinical group reported mild to moderate levels of depression (40, 41). This could have limited the statistical power and correlational analyses found with fatigue impact. In addition, this exploratory study does not present any information regarding causality in the fatigue to anhedonia pathway. Indeed, it is conceivable that anhedonia could cause disengagement in social activities leading to worsening fatigue due to deconditioning, however such conclusions would require longitudinal studies. Once again, we used the anhedonia/asociality subscale of the SANS to measure anhedonia. Indeed this subscale measures both anhedonia in terms of reduced experienced pleasure derived from activities and from social relationships. The composite score re-groups these two concepts, and a majority of studies use the total subscale score as the measure of anhedonia (32). Hence, our results do not allow for any interpretations regarding types of anhedonia. Furthermore, another limitation is that fatigue impact was assessed only using a self-evaluated measure. Indeed, individuals diagnosed with schizophrenia were historically considered as having inaccurate ratings of their own functioning and limitation (70, 71). However, recent research shows high concordance between self-reported functioning and informant reports in a sample of clinically stable individuals diagnosed with schizophrenia (72). Similarly, using the BIRT Motivation Questionnaire Raffard et al. (73) demonstrated high self-assessment reliability, in a population diagnosed with schizophrenia, in measuring their own symptoms of avolition. In addition, previous studies have also concluded that individuals diagnosed with schizophrenia have at least partial insight unto their own NS (74, 75). Hence, even though stable individuals with a diagnosis of schizophrenia may accurately evaluate their own symptomatology, future studies may benefit from including both clinician and self-rated measures of fatigue and its functional limitations. Finally, we excluded participants with any substance-use dependencies except for cannabis or tobacco. Currently there are mixed results suggesting links between symptoms of anhedonia and cannabis use in non-schizophrenic populations (76, 77). Approximately every fourth patient with a diagnosis of schizophrenia has a comorbid diagnosis of cannabis use disorder (78). Indeed, the risk of excluding people with a cannabis use disorder would be to have a non-representative sample of patients with schizophrenia. Nevertheless, future studies may benefit in controlling for the effect of cannabis use when investigating associations between negative symptomatology and experiences of fatigue in this population. Together, these suggestions would give us valuable insight for further studying fatigue in schizophrenia, as this symptom remains under-researched within this clinical population.

## Conclusion

To the best of our knowledge, this exploratory study is the first to indicate an association between fatigue impact and negative symptoms in schizophrenia. Results of this study suggest that fatigue impact was highest in the physical and social role domains (and lowest in the psychological domain). There was a clear association between anhedonia and the social impact of fatigue. Most importantly, our results suggest that when controlling for depression, the impact of fatigue in the social domain accounts for ~20% of the variance of anhedonia. We recommend further research to continue the exploration of fatigue and its impact on negative symptomatology, as well as better categorization of primary and secondary NS in hopes of developing treatments targeting specific triggers for secondary NS. Research shows that there is large functional impact due to fatigue, and that this trans-diagnostic symptom may exacerbate symptoms in schizophrenia; hence, future therapeutic interventions should target and test the efficacy of better fatigue management within this pathology in hopes of alleviating symptom burden.

## Data availability statement

The raw data supporting the conclusions of this article will be made available by the authors, without undue reservation.

## Ethics statement

The studies involving human participants were reviewed and approved by Hospital's Institutional Review Board. The patients/participants provided their written informed consent to participate in this study.

## Author contributions

SR and DC designed the study. YL and AD participated in the recruitment process. YL recruited and collected the data. YL, SR, and SB analyzed and interpreted the data. All authors contributed to the article and approved the submitted version.

## Conflict of interest

The authors declare that the research was conducted in the absence of any commercial or financial relationships that could be construed as a potential conflict of interest.

## Publisher’s note

All claims expressed in this article are solely those of the authors and do not necessarily represent those of their affiliated organizations, or those of the publisher, the editors and the reviewers. Any product that may be evaluated in this article, or claim that may be made by its manufacturer, is not guaranteed or endorsed by the publisher.
